# lncRNA myocardial infarction-associated transcript (MIAT) knockdown alleviates LPS-induced chondrocytes inflammatory injury via regulating miR-488-3p/sex determining region Y-related HMG-box 11 (SOX11) axis

**DOI:** 10.1515/biol-2021-0023

**Published:** 2021-05-31

**Authors:** Weiwei Pan, Haibao Wang, Jianwei Ruan, Wenbiao Zheng, Fanghu Chen, Jinsong Kong, Yong Wang

**Affiliations:** Department of Orthopaedic, Taizhou Municipal Hospital, No. 381, Zhongshan East Road, Jiaojiang District, Taizhou 318000, Zhejiang, China

**Keywords:** osteoarthritis, lncRNA MIAT, miR-488-3p, SOX11, inflammatory injury

## Abstract

Long noncoding RNA (lncRNA) has been shown to be involved in the development of osteoarthritis (OA), an age-related bone and joint disease. However, the function and possible molecular mechanism of lncRNA myocardial infarction-associated transcript (MIAT) in lipopolysaccharide (LPS)-induced chondrocytes injury model remain unexplored. Cell viability and apoptosis were detected by methyl thiazolyl tetrazolium (MTT) and flow cytometry, respectively. Western blot was used to detect protein expression. The concentrations of inflammatory factors were estimated by enzyme-linked immunosorbent assay (ELISA). Abundances of MIAT, microRNA-488-3p (miR-488-3p), and sex determining region Y-related HMG-box 11 (SOX11) were examined by quantitative reverse transcriptase polymerase chain reaction (qRT-PCR). Dual-luciferase reporter and RNA immunoprecipitation (RIP) assays were performed to analyze the interaction between miR-488-3p and MIAT or SOX11. LPS caused chondrocytes injury by reducing cell activity and increasing apoptosis rate and inflammatory factor secretions. Higher levels of MIAT and SOX11 and lower miR-488-3p were observed in LPS-treated C28/I2 cells. Importantly, knockdown of MIAT attenuated the LPS-induced cell injury by targeting miR-488-3p, and miR-488-3p overexpression weakened the LPS-induced cell injury by targeting SOX11. Additionally, repression of MIAT inactivated the LPS-induced NF-κB signaling pathway by decreasing SOX11 and increasing miR-488-3p. Knockdown of MIAT alleviated the LPS-induced chondrocytes injury by inhibiting the NF-κB signaling pathway mediated by the miR-488-3p/SOX11 axis.

## 1 Introduction

Osteoarthritis (OA) is a degenerative joint disease caused by obesity, aging, strain, inflammation, and trauma [1]. The common symptoms of OA patients are joint deformity, body stiffness, and movement disorder, and its incidence is associated with aging [2]. According to statistics, approximately 190 million people are suffering from OA globally, of whom 63% are over 60 years old [3]. It should be noted that the current treatment technology cannot completely cure OA, only alleviate and control its symptoms. Therefore, finding effective strategies to treat OA remains a great challenge.

Chondrocytes are the only cell species closely related to cartilage structure and function, and abnormal changes in cell vitality, apoptosis, and inflammatory response can lead to chondrocytes injury, leading to OA [4,5]. Lipopolysaccharide (LPS) has been widely reported to induce inflammatory injury of chondrocytes, which is closely related to the pathogenesis of OA [6]. Therefore, the research on the molecular mechanism of LPS-induced chondrocytes injury is of great significance for the development of targeted drugs to improve OA.

Long noncoding RNAs (lncRNAs) bear no protein-coding ability and are over 200 nucleotides long [7]. Recently, increasing lncRNAs have been found to be implicated in the pathological process of OA [8]. A report demonstrated that lncRNA GAS5 might alleviate LPS-induced chondrocytes inflammatory damage by inactivation of NF-κB and Notch signaling pathways [9]. Li et al. revealed that lncRNA PMS2L2 abated the inhibition of LPS on cell activity and promoting effects on cell apoptosis and inflammatory factors, thus protecting chondrocytes [10]. As an oncogene, lncRNA myocardial infarction-associated transcript (MIAT) is widely reported to be overexpressed in lung cancer [10], papillary thyroid cancer [11], and acute myeloid leukemia [12]. In addition, Sun et al. found that MIAT could increase the levels of inflammatory factors interleukin-1 beta (IL-1β), IL-6, and tumor necrosis factor-alpha (TNF-α) through activating the PI3K/Akt signaling pathway, thus aggravating atherosclerosis in rats [13]. Given the role of LPS in inflammatory responses, we wondered if MIAT might be participated in LPS-stimulated inflammatory injury in OA.

A large number of microRNAs (miRNAs) have been shown to be involved in LPS-induced chondrocytes damage in OA, such as miR-195-5p [14], miR-223 [15], and miR-93 [16]. At last, miR-488-3p was identified to be targeted by lncRNA PVT1 to modulate chondrocytes apoptosis [17], and it is unclear whether miR-488-3p plays a role in the inflammatory response of chondrocytes. Sex determining region Y-related HMG-box 11 (SOX11) belongs to the SOX family and is a structurally related transcription factor [18]. The effect of SOX11 was two-sided. It seemed to be determined by the type of cancers, both carcinogenic [19] and anticancer [20]. Proteomics analysis by Iliopoulos et al. revealed that SOX11 was differentially expressed in osteoarthritic cartilage and normal cartilage [21], and SOX11 could improve the level of pro-inflammatory factor TNF-α [22]. However, the connection between miR-488-3p and SOX11 and their underlying mechanism in OA has not been revealed.

In general, this paper explored the function of MIAT and the molecular mechanism mediated by it in LPS-induced chondrocytes injury models.

## 2 Materials and methods

### 2.1 Tissue samples

Knee cartilage and normal tissues (control) were collected from 30 OA patients and 30 healthy participants at Taizhou Municipal Hospital, respectively. The clinicopathological data of the patients were exhibited in Table 1. All tissues were stored in liquid nitrogen.

**Table 1:** The clinicopathological data of the patients involved in the study

Clinicopathological features		
Total = 60		
Group	OA (*n* = 30)	Control (*n* = 30)
Mean age (years)	56.3 ± 3.6	51.2 ± 2.9
Gender (males/females)	12/18	14/16
OA stage	—	—
Stage I	12	
Stage II	7	
Stage III	8	
Stage IV	3	


**Informed consent:** Informed consent has been obtained from all individuals included in this study.
**Ethical approval:** The research related to human use has been complied with all the relevant national regulations, institutional policies, and in accordance with the tenets of the Helsinki Declaration and has been approved by the Ethics committee of Taizhou Municipal Hospital.

### 2.2 Cell culture and LPS stimulation

Human normal cartilage cell line C28/I2 was obtained by BeNa Culture Collection (Beijing, China). ATDC5 was purchased by American Type Culture Collection (ATCC, Manassas, VA, USA). Both cell lines were cultured in Dulbecco’s modified Eagle’s medium-F12 (DMEM-F12, Gibco, Carlsbad, CA, USA) with 10% fetal bovine serum (FBS, Gibco) at 37°C with 5% CO_2_. For LPS stimulation, C28/I2 cells were exposed to various doses of LPS (1, 5 and 10 μg/mL) for 12 h, and the untreated cells were the control groups.

### 2.3 Transfection

Small interfering RNAs-targeted MIAT (si-MIAT) and si-NC were obtained from GenePharma (Shanghai, China). Additionally, miR-488-3p mimic (miR-488-3p), inhibitor (anti-miR-488-3p), and the corresponding controls (miR-NC, anti-miR-NC) were also acquired from GenePharma. MIAT full-length and SOX11 coding sequence (CDS) were cloned into the pcDNA vector (Invitrogen, Carlsbad, CA, USA) to form MIAT and SOX11 overexpressed plasmids, respectively. The empty vector pcDNA was employed as their control. These constructs were transfected into C28/I2 cells by using Lipofectamine 3000 (Invitrogen).

### 2.4 Quantitative reverse transcriptase polymerase chain reaction (qRT-PCR)

The RNA from knee cartilage tissues and C28/I2 cells was extracted by TRIzol^®^ reagent (Invitrogen), and the complementary DNA (cDNA) was synthesized by SuperScript™ IV Reverse Transcriptase (Thermo Fisher Scientific, Waltham, MA, USA). SYBR Green PCR Master Mix (Applied Biosystems, Foster City, CA, USA) was used to perform qRT-PCR and all primers were synthesized by GenePharma. Glyceraldehyde-3-phosphate dehydrogenase (GAPDH) was used as the control for MIAT and SOX11, and miR-488-3p expression was normalized to U6. Primer sequences: MIAT (F, 5′-TTTACTTTAACAGACCAGAA-3′, R, 5′-CTCCTTTGTTGAATCCAT-3′). GAPDH (F, 5′-TATGATGATATCAAGAGGGTAGT-3′, R, 5′-TGTATC CAAACTCATTGTCATAC-3′). miR-488-3p (F, 5′-CGGGGCAGCUCAGUACAG-3′, R, 5′-CAGTGCGTGTCGTGGAGT-3′). U6 (F, 5′-GTGCGTGTCGTGGAGTCG-3′, R, 5′-AACGCTTCACGAATTTGCGT-3′). SOX11 (F, 5′- GGTGGATAAGGATTTGGATTCG-3′, R, 5′-GCTCCGGCGTGCAGTAGT-3′). Results were calculated using 2^−ΔΔCt^ method.

### 2.5 Cell viability and apoptosis assay

C28/I2 cells were seeded on 96-well plates and were stimulated with different doses of LPS (1, 5 and 10 μg/mL) for 12 h. After that, cells were incubated with 20 µL of Methyl thiazolyl tetrazolium (MTT, Thermo Fisher Scientific) solution for 4 h at 37°C. After dissolving the formazan crystals, the absorbance was measured by a Microplate Reader (Bio-Rad, Hercules, CA, USA) at 490 nm.

Apoptosis analysis was performed by an Annexin V fluorescein isothiocynate (FITC) and propidium iodide (PI) apoptosis detection kit (BD Biosciences, Franklin Lakes, NJ, USA) using Flow cytometry. C28/I2 cells were stimulated by LPS or transfected for 48 h. Then cells were harvested and stained with 5 µL V-FITC as well as 5 µL PI for 20 min in the absence of light. Subsequently, cell apoptosis was determined by a flow cytometer (BD Biosciences).

### 2.6 Western blot

After the treated C28/I2 cells were harvested, 30 μg of protein was extracted by RIPA lysis buffer (Beyotime, Shanghai, China), separated on SDS-PAGE gel and then transferred to polyvinylidene difluoride (PVDF, Millipore, Bedford, MA, USA) membranes. Subsequently, the membranes were immersed in 5% nonfat milk for 2 h and incubated with primary antibodies against B cell lymphoma-2 (Bcl-2, 1:1,000, Abcam, Cambridge, MA, USA), Bcl-2-associated X protein (Bax, 1:2,000, Abcam), SOX11 (1:2,000, Abcam), phosphorylation-P65 (p-P65, 1:1,000, Thermo Fisher Scientific), P65 (5 µg/mL, Thermo Fisher Scientific), IkB-α (1:2,000, Abcam), p-IkB-α (1:3,000, Abcam), Toll-like receptor 4 (TLR4, 1:1,000, Abcam), or GAPDH (1:3,000, Abcam) at 4°C overnight. The membranes were then probed with a secondary antibody conjugated with horseradish peroxidase (1:5,000, Abcam) for 1 h at 37°C. The blots were visualized by RapidStep ECL Reagent (Millipore Corp., Billerica, MA, United States), and the results were observed using Bio-Rad ChemiDoc XRS + Chemiluminescence Imaging System.

### 2.7 Enzyme-linked immunosorbent assay (ELISA)

C28/I2 cells were seeded into the 24-well plates. Cells were treated with or without 10 μg/mL LPS and the culture supernatant was collected at 48 h after transfection. The concentrations of inflammatory cytokines IL-8 (PI640), interleukin-1 beta (IL-1β) (PI305), and TNF-α (PT518) were examined by the ELISA kit (Beyotime).

### 2.8 Dual-luciferase reporter assay

The fragments from wild-type MIAT and SOX11 with miR-488-3p binding sites were inserted into a pmirGLO report vector (Promega, Madison, WI, USA) to form WT-MIAT and SOX1 3′UTR-WT report vectors, respectively. Mutant-MIAT (MUT-MIAT) and SOX1 3′UTR-WT report vectors without miR-488-3p binding sites were built in the same way. These report vectors were co-transfected with miR-488-3p or miR-NC into C28/I2 cells using Lipofectamine 3000 (Invitrogen) for 24 h. The luciferase activity was determined by a dual-luciferase reporter assay kit (Promega), according to the manufacturers’ instructions.

### 2.9 RNA immunoprecipitation (RIP) assay

EZ-Magna RIP Kit (Millipore) was used in RIP assay. C28/I2 cells were treated with the lysis buffer and incubated with magnetic beads. Later, the beads were washed with 500 μL RIP Wash Buffer before hatching with Argonaute2 (Ago2) or ImmunoglobulinG (IgG) antibody. Finally, the RNAs bound to the magnetic beads were purified and extracted. Abundances of MIAT, miR-488-3p, and SOX11 were analyzed by qRT-PCR as described above.

### 2.10 Statistical analysis

SPSS 19.0 software was used to analyze the data, which were shown as the mean ± standard deviation (SD) of at least 3 independent experiments. The *p-*value was obtained via Student’s *t*-test between two groups and one-way analysis of variance (ANOVA) among multiple groups, and its value of less than 0.05 was considered a significant difference.

## 3 Results

### 3.1 LPS-induced inflammatory injury cell model was successfully constructed

**Figure 1:**
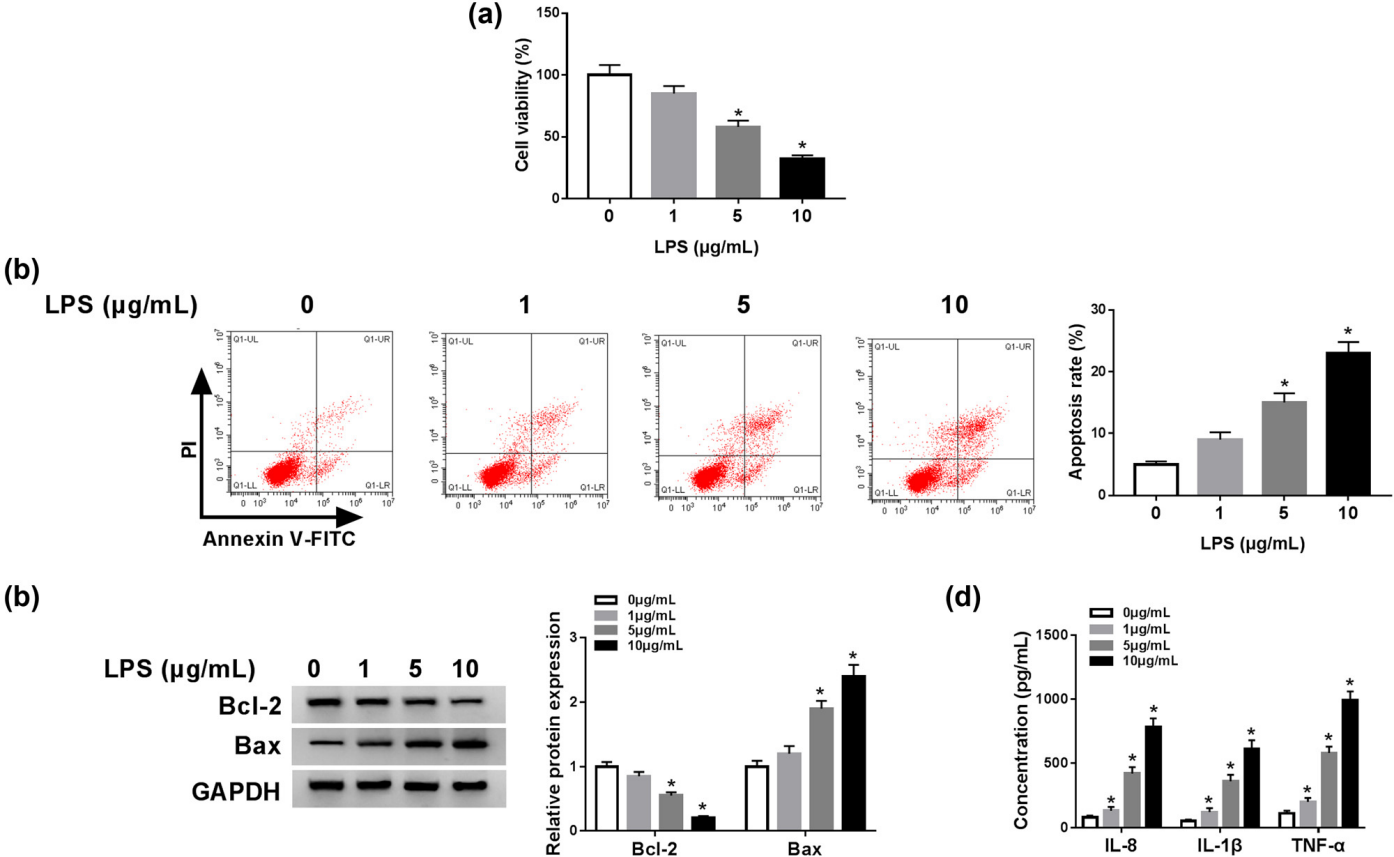
LPS-induced inflammatory injury cell model was successfully constructed. C28/I2 cells were induced by various doses of LPS (0, 1, 5, and 10 μg/mL). (a) Cell viability was estimated by MTT assay. (b) Cell apoptosis was evaluated by Flow cytometry. (c) The expression levels of apoptosis-associated proteins Bcl-2 and Bax were detected by western blot analysis. (d) The concentrations of inflammatory factors IL-8, IL-1β, and TNF-α were measured by ELISA assay. *P < 0.05.

As displayed in Figure 1a, cell viability was evidently reduced by treatment of 5 μg/mL LPS and 10 μg/mL LPS. Flow cytometry results showed that LPS substantially increased the apoptosis rate of C28/I2 cells at the concentrations of 5 and 10 μg/mL compared to untreated cells (Figure 1b). Western blot data indicated that the expression of antiapoptotic Bcl-2 was significantly decreased, while the expression of proapoptotic Bax and Caspase 11 was strikingly increased after LPS stimulation (5 and 10 μg/mL) in C28/I2 cells (Figure 1c and Figure S2). Furthermore, ELISA assay revealed that the concentrations of IL-8, IL-1β, and TNF-α were enormously enhanced in LPS-treated (1, 5, and 10 μg/mL) C28/I2 cells than untreated cells (Figure 1d). These results proved that the model of LPS-induced C28/I2 cells inflammatory injury was successfully constructed.

### 3.2 MIAT was upregulated and miR-488-3p was downregulated in OA tissues and LPS-stimulated chondrocytes

**Figure 2:**
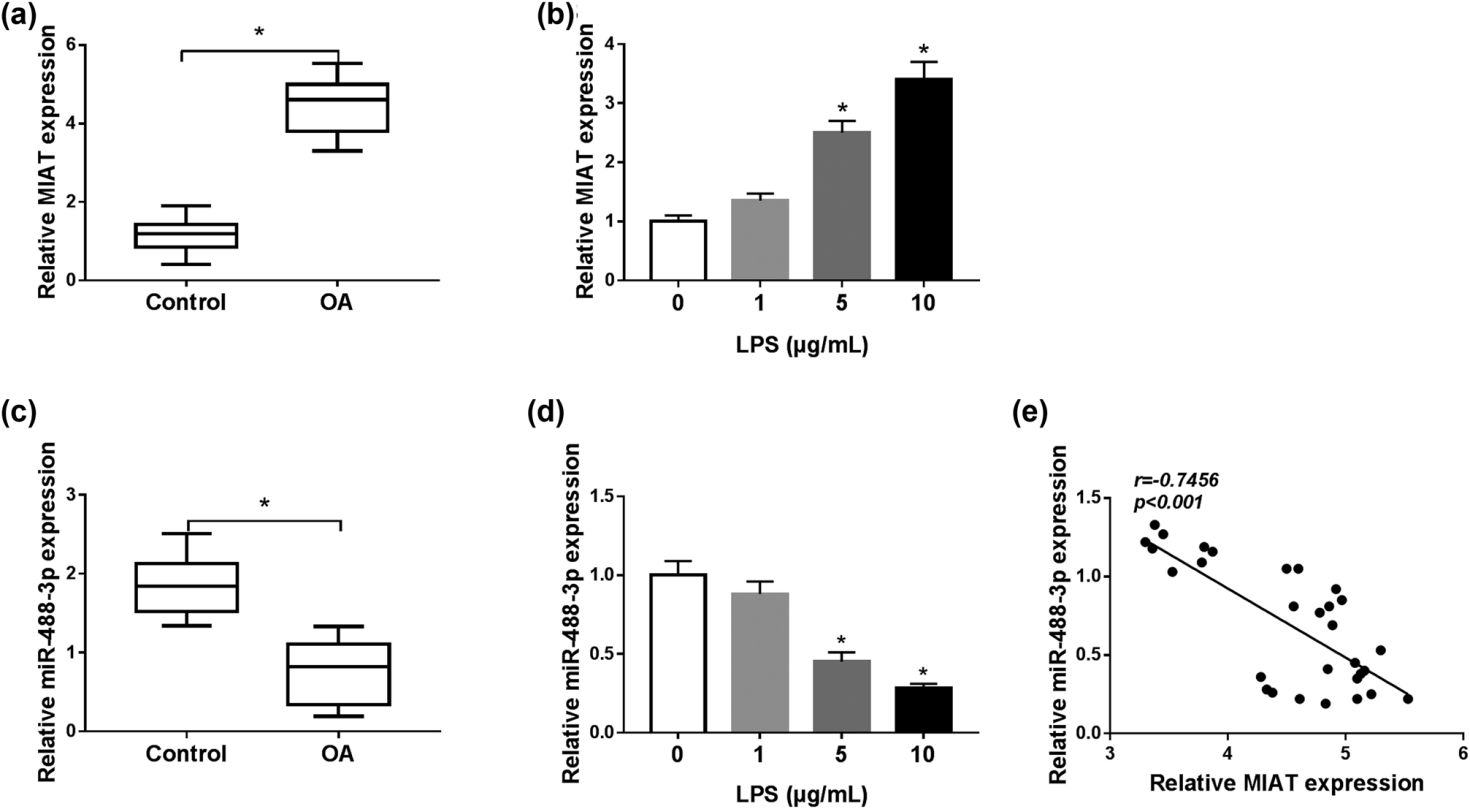
MIAT was upregulated and miR-488-3p was downregulated in OA tissues and LPS-stimulated chondrocytes. (a) MIAT expression in OA and normal tissues was measured by qRT-PCR. (b) C28/I2 cells were treated by various doses of LPS, and MIAT expression was determined using qRT-PCR analysis. (c) miR-488-3p expression in OA and normal tissues was measured by qRT-PCR. (d) miR-488-3p expression in different doses of LPS-treated C28/I2 cells was examined by qRT-PCR. (e) Pearson correlation coefficient was used to analyze the relationship between MIAT expression and miR-488-3p expression in OA tissues. *P < 0.05.

To understand whether the expression of MIAT was altered in patients with OA, we measured MIAT expression by qRT-PCR. As shown in Figure 2a, MIAT expression was upregulated in OA tissues compared with the control group. We also detected its expression in LPS-stimulated chondrocytes and found that MIAT expression was significantly upregulated in C28/I2 cells by stimulation of LPS at the concentrations of 5 and 10 μg/mL in contrast to untreated cells (Figure 2b). Just the opposite, miR-488-3p expression was drastically downregulated in OA tissues (Figure 2c) and LPS-induced (5 and 10 μg/mL) C28/I2 cells (Figure 2d). Notably, a significant inverse correlation between MIAT expression and miR-488-3p expression in OA tissues was analyzed (Figure 2e). The findings supported that MIAT and miR-488-3p might play important roles in the development of OA.

### 3.3 LPS-induced injury could be partially weakened by MIAT knockdown

**Figure 3:**
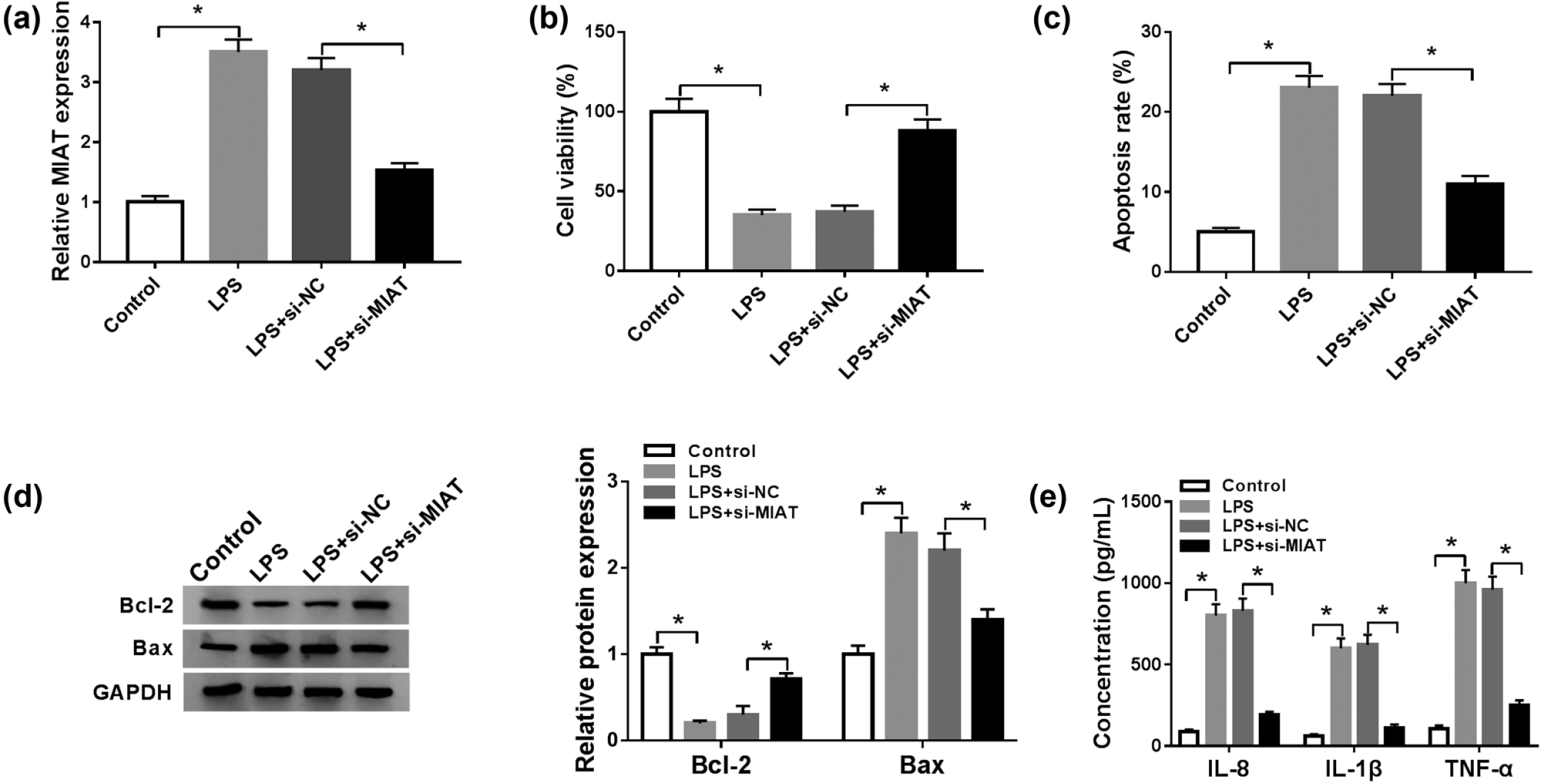
LPS-induced injury could be partially weakened by MIAT knockdown. C28/I2 cells were divided into the following four groups: control, LPS, LPS + si-NC, and LPS + si-MIAT. (a) MIAT expression was measured by qRT-PCR. (b and c) Cell viability and apoptosis were evaluated by MTT assay and Flow cytometry, respectively. (d) The protein levels of Bcl-2 and Bax were assessed by western blot. (e) The concentrations of IL-8, IL-1β, and TNF-α were examined by ELISA assay. *P < 0.05.

To evaluate the effect of MIAT on LPS-induced (10 μg/mL) inflammatory injury, we knocked down MIAT by siRNA in LPS-treated C28/I2 cells. As shown in Figure 3a, MIAT was markedly declined by transfection of si-MIAT compared to cells transfected with si-NC. Subsequently, MTT assay showed that the repression of MIAT rescued the inhibition of LPS on cell viability (Figure 3b). Meanwhile, the promoting effect of LPS on cell apoptosis was also reversed by MIAT knockdown (Figure 3c). Besides, transfection of si-MIAT in LPS-treated C28/I2 cells increased Bcl-2 expression and silenced Bax expression (Figure 3d). ELISA assay results demonstrated that the concentrations of IL-8, IL-1β, and TNF-α were decreased by silencing MIAT in LPS-stimulated cells (Figure 3e). All the results indicated that knockdown of MIAT could alleviate the LPS-induced chondrocytes injury.

### 3.4 MIAT knockdown alleviated the LPS-induced chondrocytes injury by targeting miR-488-3p

**Figure 4:**
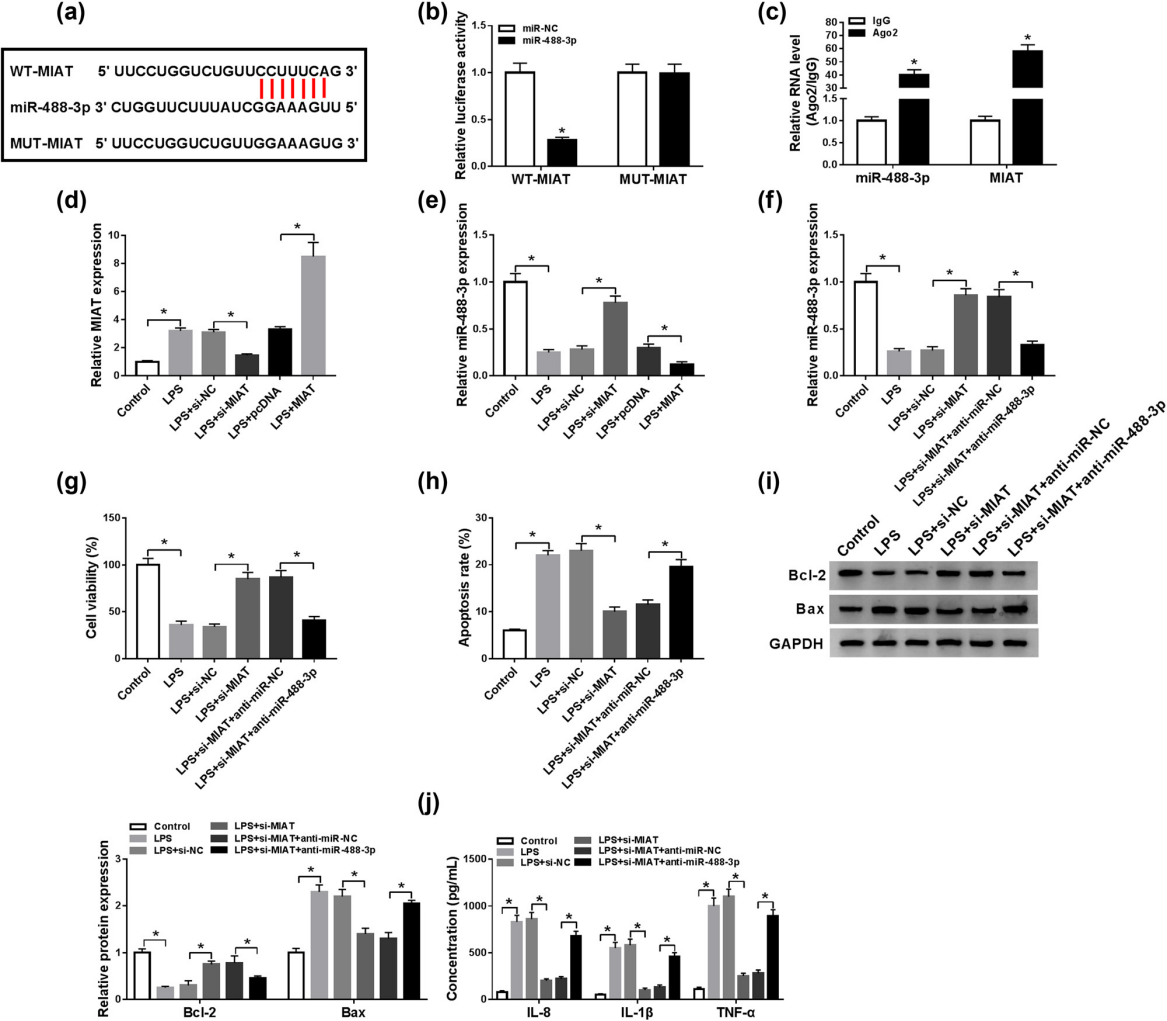
MIAT knockdown alleviated the LPS-induced chondrocytes injury by targeting miR-488-3p. (a) StarBase v2.0 predicted that there were binding sites between MIAT and miR-488-3p. (b and c) Dual-luciferase reporter and RIP assays were conducted to identify the combination of MIAT and miR-488-3p. (d and e) LPS-treated C28/I2 cells were transfected with si-NC, si-MIAT, pcDNA, or MIAT, and the levels of MIAT and miR-488-3p were measured by qRT-PCR. (f) C28/I2 cells were exposed to different treatments (control, LPS, LPS + si-NC, LPS + si-MIAT, LPS + si-MIAT + anti-miR-NC or LPS + si-MIAT + anti-miR-488-3p), miR-488-3p expression was then detected by qRT-PCR. (g) Cell viability was then assessed by MTT assay. (h) Cell apoptosis was then analyzed by Flow cytometry. (i) Western blot assay was used to measure the levels of Bcl-2 and Bax. (j) ELISA assay was utilized to evaluate the concentrations of IL-8, IL-1β, and TNF-α. *P < 0.05.

Considering the negative correlation between MIAT and miR-488-3p in OA tissues and their opposite expression patterns in LPS-treated chondrocytes, we then explored their relationship in OA. Coincidentally, there were complementary sites between MIAT and miR-488-3p (Figure 4a). Dual-luciferase reporter assay showed that the luciferase activity was steeply decreased in cells co-transfected with WT-MIAT and miR-488-3p than that in the cells co-transfected with MUT-MIAT and miR-488-3p (Figure 4b). Additional RIP experiments showed that the levels of MIAT and miR-488-3p were aggrandized in C28/I2 cells incubated with Ago2 antibody compared to the control group (Figure 4c). The above results suggested that MIAT can specifically bind to miR-488-3p. Moreover, qRT-PCR results showed that MIAT knockdown augmented miR-488-3p expression in LPS-treated C28/I2 cells, and MIAT overexpression degraded miR-488-3p expression (Figure 4d and e). To figure out whether MIAT mediated LPS-induced chondrocytes injury by regulating miR-488-3p, si-MIAT and anti-miR-488-3p were co-transfected into LPS-treated C28/I2 cells. As displayed in Figure 4f, interference with miR-488-3p could reverse the promoting effect of si-MIAT on miR-488-3p expression. MTT and Flow cytometry data showed that the effects of si-MIAT on cell viability (Figure 4g) and apoptosis (Figure 4h) were overturned by co-transfection with anti-miR-488-3p in LPS-treated C28/I2 cells. Likewise, the effects of si-MIAT on expression levels of apoptotic proteins Bcl-2 and Bax (Figure 4i) and the concentrations of inflammatory factors IL-8, IL-1β, and TNF-α (Figure 4j) could also be neutralized by suppression of miR-488-3p. To sum up, MIAT knockdown alleviated the LPS-induced chondrocytes injury by upregulating miR-488-3p.

### 3.5 miR-488-3p directly interacted with SOX11

**Figure 5:**
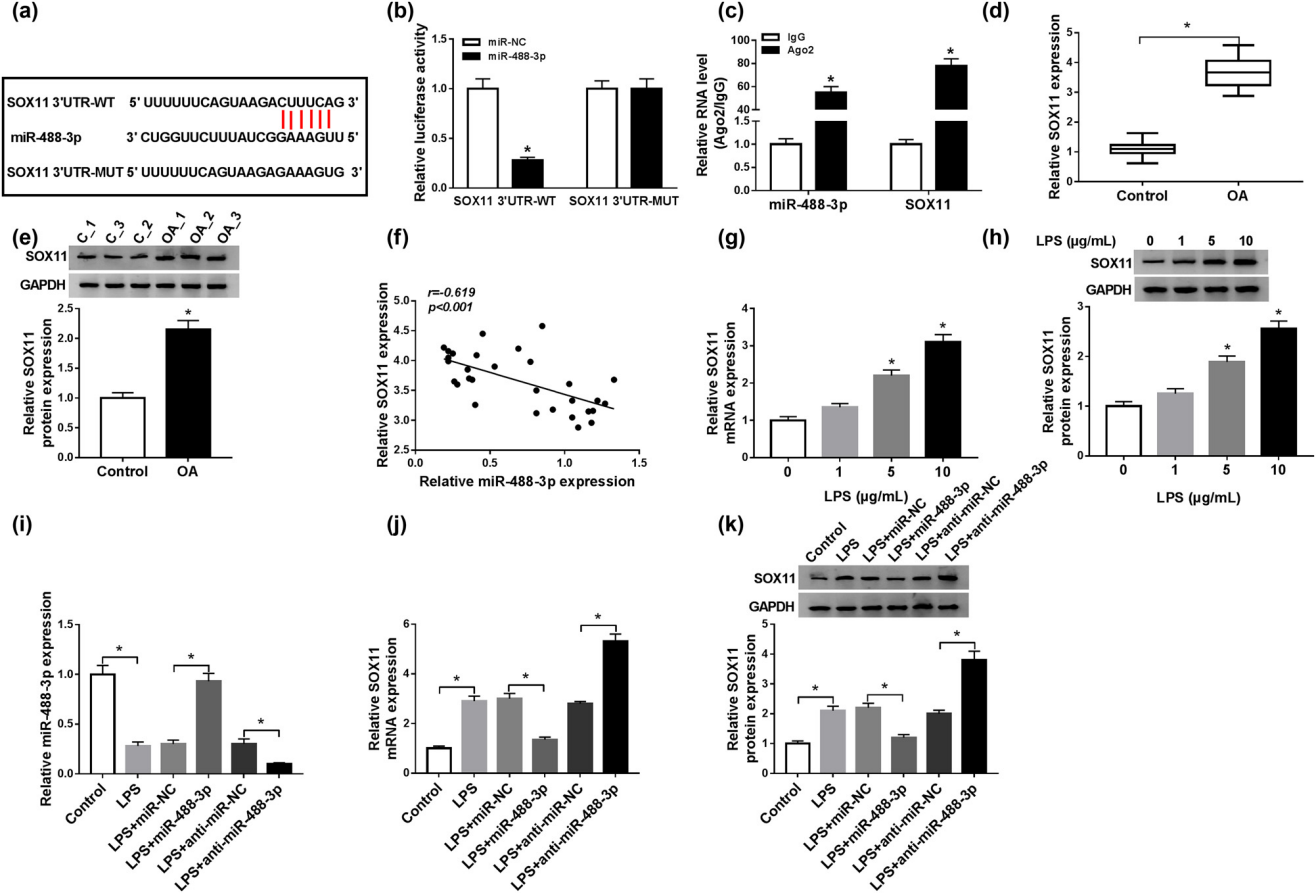
miR-488-3p directly interacted with SOX11. (a) StarBase v2.0 predicted that there were binding sites between miR-488-3p and SOX11. (b and c) Dual-luciferase reporter and RIP assays were performed to notarize the interaction between miR-488-3p and SOX11. (d and e) The mRNA and protein expression levels of SOX11 were detected in OA tissues by qRT-PCR and western blot, respectively. (f) The correlation between SOX11 expression and miR-488-3p expression in OA tissues was analyzed by Pearson correlation coefficient. (g and h) The mRNA and protein expression levels of SOX11 in C28/I2 cells treated with various doses of LPS (0, 1, 5, and 10 μg/mL) were examined by qRT-PCR and western blot, respectively. (i–k) C28/I2 cells were exposed to different treatments (control, LPS, LPS + miR-NC, LPS + miR-488-3p, LPS + anti-miR-NC or LPS + anti-miR-488-3p). (i) miR-488-3p expression was detected by qRT-PCR. (j and k) The mRNA and protein expression levels of SOX11 were then detected by qRT-PCR and western blot, respectively. *P < 0.05.

To further explore the downstream mechanism of MIAT/miR-488-3p, we used starBase v2.0 bioinformatics analysis to search for the target genes of miR-488-3p. It was found that there were binding sites between miR-488-3p and SOX11 (Figure 5a). Similarly, dual-luciferase reporter assay and RIP assay were employed. The results demonstrated that miR-488-3p could distinctly reduce the luciferase activity of SOX1 3′UTR-WT; however, no significant change was observed in the luciferase activity of SOX1 3′UTR-MUT (Figure 5b). RIP assay results showed that the adsorption level of Ago2 antibody to miR-488-3p and SOX11 was higher than that of IgG antibody (Figure 5c). These results supported that SOX11 was a target of miR-488-3p. Meanwhile, we examined the expression of SOX11 in OA. As presented in Figure 5d and e, the mRNA and protein expression levels of SOX11 were raised in OA tissues. As expected, miR-488-3p expression was negatively associated with SOX11 expression (Figure 5f). Furthermore, the mRNA and protein expression levels of SOX11 were distinctly elevated after LPS stimulation (5 and 10 μg/mL) in C28/I2 cells (Figure 5g and h). The effect of miR-488-3p on SOX11 expression in LPS-stimulated C28/I2 cells was then studied. The miR-488-3p overexpression and interference efficiency were first detected by qRT-PCR (Figure 5i). Overexpression of miR-488-3p decreased the mRNA and protein levels of SOX11, and miR-488-3p knockdown enhanced SOX11 expression at mRNA and protein levels (Figure 5j and k). The above results revealed that miR-488-3p directly targeted SOX11 and negatively modulated SOX11 expression.

### 3.6 Upregulation of miR-488-3p alleviated the LPS-induced chondrocytes injury by inhibiting SOX11

**Figure 6:**
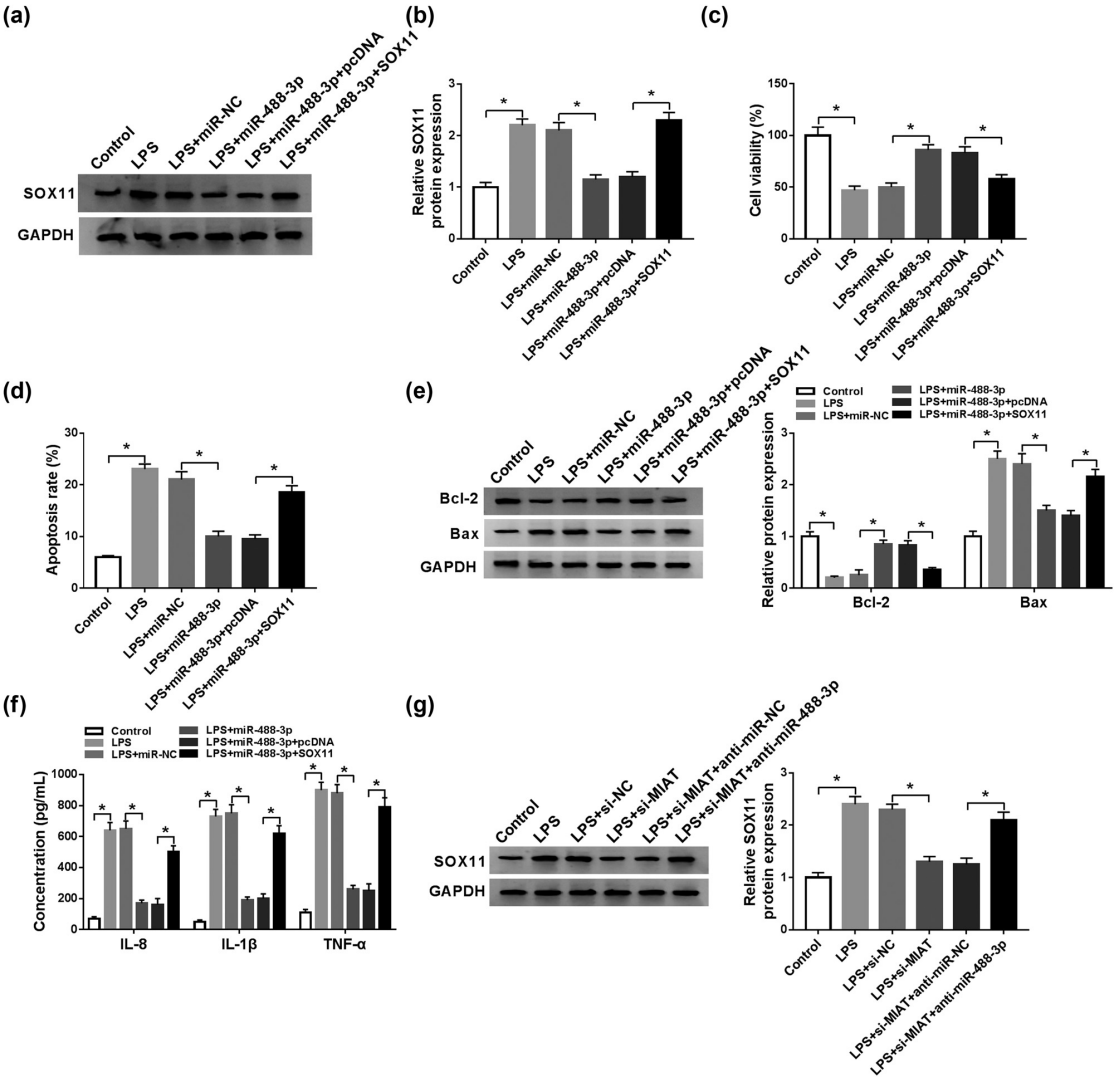
Upregulation of miR-488-3p alleviated the LPS-induced chondrocytes injury by inhibiting SOX11. C28/I2 cells were exposed to different treatments (control, LPS, LPS + miR-NC, LPS + miR-488-3p, LPS + miR-488-3p + pcDNA or LPS + miR-488-3p + SOX11). (a and b) The protein expression of SOX11 was detected by western blot. (c and d) Cell viability and apoptosis were assessed by MTT assay and Flow cytometry, respectively. (e) The levels of Bcl-2 and Bax were checked by western blot. (f) ELISA assay was used to measure the concentrations of IL-8, IL-1β, and TNF-α. (g) C28/I2 cells were exposed to different treatments (control, LPS, LPS + si-NC, LPS + si-MIAT, LPS + si-MIAT + anti-miR-NC or LPS + si-MIAT + anti-miR-488-3p), SOX11 expression was then examined by western blot. *P < 0.05.

As appeared in Figure 6a and b, after co-transfection of SOX11 in LPS-treated C28/I2 cells, the inhibitory effect of miR-488-3p overexpression on SOX11 protein expression was reversed. In addition, after overexpression of miR-488-3p in LPS-induced C28/I2 cells, cell viability was potentiated (Figure 6c), cell apoptosis rate was restrained (Figure 6d), Bcl-2 expression was increased, Bax expression was decreased (Figure 6e), and the concentrations of IL-8, IL-1β, and TNF-α were repressed (Figure 6f). Still, these effects were abolished by overexpressing SOX11. Additionally, repression of MIAT could reduce the protein expression of SOX11, while miR-488-3p depletion could weaken the MIAT effect on SOX11 expression (Figure 6g and Figure S1). Overexpression of SOX11 reversed the effect of miR-488-3p on LPS-induced chondrocytes injury, and MIAT could act as miR-488-3p sponge to mediate SOX11 expression.

### 3.7 Silencing MIAT could inactivate the LPS-induced NF-κB signaling pathway by regulating miR-488-3p/SOX11 axis

**Figure 7:**
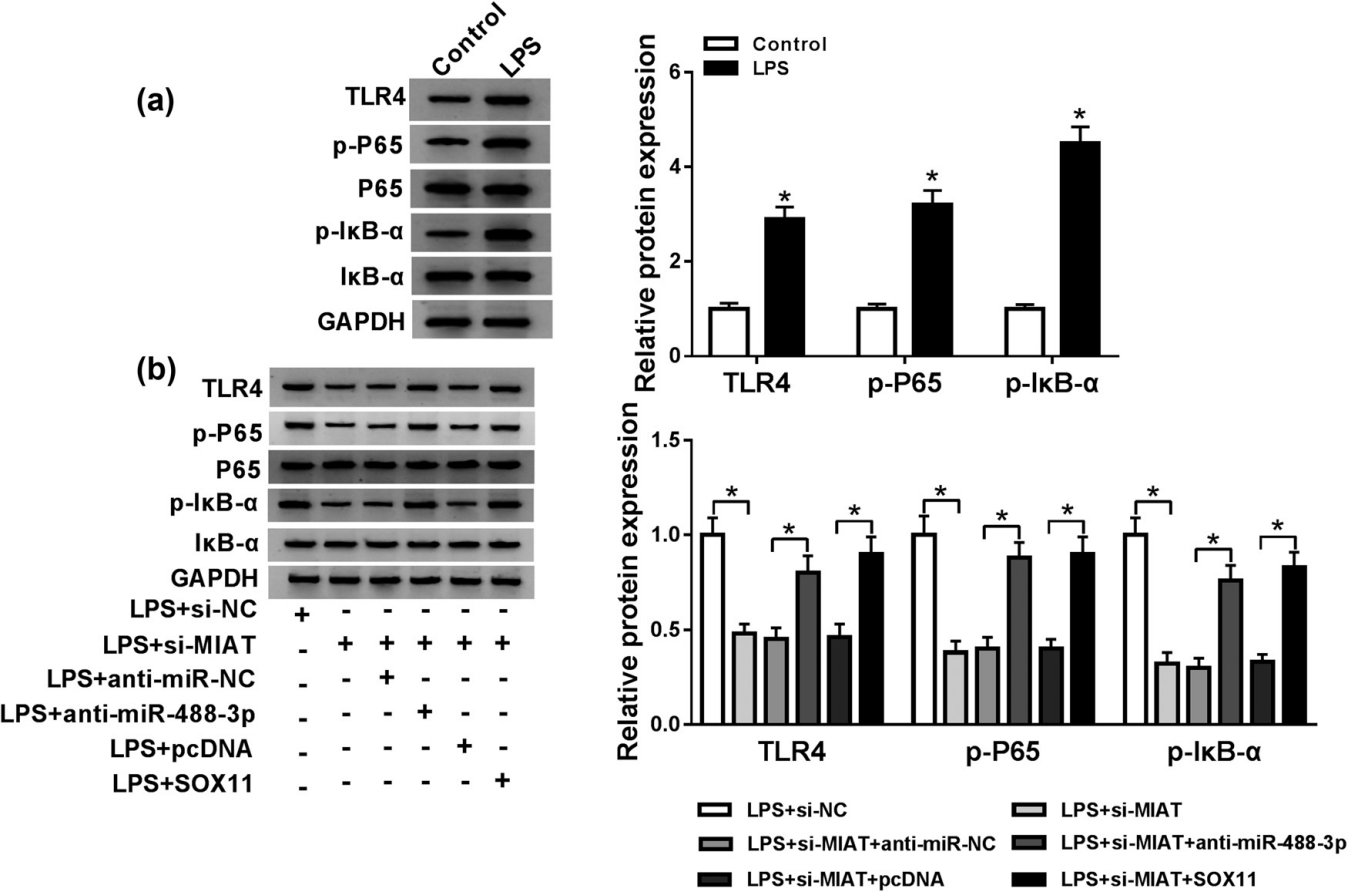
Silencing MIAT could inactivate the LPS-induced NF-κB signaling pathway by regulating miR-488-3p/SOX11 axis. (a) The expression levels of TLR4, p-P65, P65, IkB-α, and p-IkB-α in C28/I2 cells treated with LPS or control were detected by western blot. (b) LPS induced C28/I2 cells were transfected with si-NC, si-MIAT, si-MIAT + anti-miR-NC, si-MIAT + anti-miR-488-3p, si-MIAT + pcDNA or si-MIAT + SOX11, respectively. And the protein levels of TLR4, p-P65 and p-IkB-α were determined by western blot.

Previous studies have shown that LPS could activate the NF-κB signaling pathway. We then detected the effect of LPS on NF-κB signaling pathway. As shown in Figure 7a, the levels of phosphorylated proteins p-P65 and p-IkB-α were significantly increased in LPS-treated C28/I2 cells and the Toll-like receptor 4 (TLR4) expression was also upregulated in LPS-treated C28/I2 cells. More than that, we found that the promotion effects of LPS on levels of TLR4, p-P65, and p-IkB-α were reversed by interfering with MIAT, and the effect of si-MIAT could be attenuated by repression of miR-488-3p or overexpression of SOX11 (Figure 7b). The results implied that MIAT knockdown inactivated the LPS-induced NF-κB signaling pathway and TLR4 expression by regulating miR-488-3p/SOX11 axis.

## 4 Discussion

As a degenerative disease that cannot be cured entirely, OA affects millions of people worldwide. There is an urgent need to explore new molecular targets for developing or improving drugs against OA. OA is considered to be an inflammatory disease leading to the degradation and injury of chondrocytes [23]. LPS has been proved to be a strong inducer of inflammatory responses. For instance, it inhibited the viability of chondrocytes ATDC5 and induced the secretion of various inflammatory factors including IL-6, IL-8, IL-1β, and TNF-α, as well as improved the cell apoptosis rate [24]. Our findings were consistent with previous studies mentioned above, suggesting that LPS strongly induced chondrocytes injury by reducing cell activity, promoting apoptosis, and releasing IL-8, IL-1β, and TNF-α, which proved that the model of LPS-induced chondrocytes injury was successfully established.

lncRNA has been reported to play a pivotal regulatory role in LPS-induced chondrocytes injury [25]. The MIAT-related research was predominantly focused on cancer, in which MIAT was validated as a promoter of tumor development [26]. Not long ago, Li et al. [27] have shown that MIAT promotes the apoptosis of LPS-induced chondrocytes and MIAT’s overexpression leads to the cytokine release. However, the research on MIAT in OA is limited, and we further explored its function and potential mechanism. Our results showed that MIAT was drastically overexpressed in OA patients and LPS-treated C28/I2 cells. Interference with MIAT increased cell activity and inhibited cell apoptosis and inflammatory reaction, indicating that the LPS-induced cell injury was largely alleviated by silencing MIAT. Our data revealed the protective effect of MIAT knockdown on chondrocytes, which was in agreement with a previous study [27].

lncRNAs can act as the sponges for miRNAs to modulate the expression of target genes [28], and MIAT could serve as a ceRNA to increase sirt1 expression by sponging miR-22-3p in hepatocellular carcinoma [29]. In this work, we indicated that the expression pattern of miR-488-3p in OA tissues and LPS-treated C28/I2 cells was opposite to MIAT and verified that miR-488-3p was the downstream target miRNA of MIAT. Furthermore, miR-488-3p suppression rescued the impact of MIAT knockdown on LPS-stimulated chondrocytes injury, which was in agreement with previous reports [17]. Additionally, Zhou et al. indicated that miR-488 was distinctly declined in peripheral white blood cells of patients with acute gouty arthritis, and miR-488 upregulation could restrain the expression of pro-inflammatory factor IL-1β [30]. Based on that we’ve concluded that MIAT depletion alleviated the LPS-induced chondrocytes injury through targeting miR-488-3p.

Subsequently, SOX11 was confirmed as a target mRNA of miR-488-3p, and its expression was augmented in LPS-induced chondrocytes. Previously, using microarray analysis, Fu et al. revealed that SOX11 was upregulated in OA cartilage versus normal samples [31], and it could positively regulate the levels of the inflammatory cytokines TLR4 and iNOS [32]. Moreover, higher levels of SOX4 and SOX11 were found in the inflamed synovium of patients with arthritis [33]. In our paper, SOX11 overexpression potentiated LPS-induced chondrocytes injury, which counteracted the mitigating impact of miR-488-3p on LPS-induced chondrocytes injury. Importantly, MIAT positively regulated SOX11 expression by acting as a ceRNA of miR-488-3p. It has been proved that NF-κB signaling pathway plays a pivotal role in OA, and its inactivation can prevent the inflammatory response of chondrocytes [34,35]. In this study, LPS stimulation significantly promoted phosphorylation of P65 and IkB-α proteins, implying possible activation of NF-κB signaling pathway, while knockdown of MIAT could inactivate said pathway by targeting miR-488-3p or regulating SOX11. Taken together, these results suggest that the NF-κB signaling pathway might be involved in the chondrocytes injury process regulated by MIAT. Furthermore, previous study showed that SOX11 could upregulate the expression level of TLR4 in spinal cord injury [36]. Therefore, we speculated that SOX11 might regulate the TLR4 levels in LPS-induced chondrocytes. The data showed that LPS enhances TLR4 expression in chondrocytes. Additionally, MIAT knockdown could downregulate the TLR4 levels, and miR-488-3p inhibition or SOX11 overexpression could restore the effects of MIAT inhibition on TLR4 expression in LPS-treated chondrocytes. So MIAT knockdown alleviates LPS-induced chondrocytes inflammatory injury via regulating miR-488-3p/SOX11 axis through NF-κB signaling pathway and regulating TLR4 expression in osteoarthritis.

To conclude, we’ve revealed a protective role of MIAT knockdown in LPS-stimulated chondrocytes inflammatory injury. This effect might be achieved through inactivation of the NF-κB signaling pathway by regulation of the miR-488-3p/SOX11 axis. However, the limitation of this study is lack of animal experiments due to the laboratory conditions. In the future, we will focus on the *in vivo* experiments, which may help to better understand the role of MIAT in OA.
